# Purified Plant-Derived Phenolic Acids Inhibit *Salmonella* Typhimurium without Alteration of Microbiota in a Simulated Chicken Cecum Condition

**DOI:** 10.3390/microorganisms11040957

**Published:** 2023-04-06

**Authors:** Zabdiel Alvarado-Martinez, Zajeba Tabashsum, Arpita Aditya, Grace Suh, Matthew Wall, Katherine Hshieh, Debabrata Biswas

**Affiliations:** 1 Biological Sciences Program-Molecular and Cellular Biology, University of Maryland-College Park, College Park, MD 20742, USA; zalvara1@umd.edu (Z.A.-M.); ztabashs@terpmail.umd.edu (Z.T.); 2 Department of Animal and Avian Sciences, University of Maryland-College Park, College Park, MD 20742, USA; aaditya1@umd.edu; 3 Department of Biology, University of Maryland-College Park, College Park, MD 20742, USA; ssgrace737@gmail.com (G.S.); mjwall@terpmail.umd.edu (M.W.); khshieh@terpmail.umd.edu (K.H.); 4 Center for Food Safety and Security Systems, University of Maryland-College Park, College Park, MD 20742, USA

**Keywords:** *Salmonella* Typhimurium, gallic acid, protocatechuic acid, vanillic acid, phenolic acids, microbiome, poultry

## Abstract

*Salmonella enterica* serovar Typhimurium (ST) remains a predominant zoonotic pathogen because of its colonization in poultry, survivability in the environment, and increasing antibiotic-resistance pattern. Plant-derived phenolics, gallic acid (GA), protocatechuic acid (PA), and vanillic acids (VA) have demonstrated antimicrobial activity in vitro; therefore, this study collected chicken cecal fluid and supplemented it with these phenolics to evaluate their potential for eliminating ST and mod-ulating the microbiota of complex environments. ST was quantified through plating, while micro-biome analysis was performed through pair-end 16S-rRNA gene sequencing. CFU/mL of ST in cecal fluid with GA was significantly reduced by 3.28 and 2.78 log at 24 h and 48 h, while PA only had a slight numerical decrease. VA significantly reduced ST by 4.81 and 5.20 log at 24 h and 48 h. Changes in relative abundance of major phyla were observed at 24 h for samples with GA and VA as Firmicute levels increased 8.30% and 20.90%, while Proteobacteria decreased 12.86% and 18.48%, respectively. Significant changes in major genre were observed in *Acinetobacter* (3.41% for GA) and *Escherichia* (13.53% for VA), while *Bifidobacterium* increased (3.44% for GA) and *Lactobacillus* remained unchanged. Results suggest that phenolic compounds exert different effects on certain pathogens, while supporting some commensal bacteria.

## 1. Introduction

The current demands for novel antimicrobial compounds, which can target and eliminate pathogenic bacteria without negative consequences, is critical as multiple antibiotic-resistant bacterial pathogens continue to be a major cause of disease, hospitalization, and death [1]. This emerging issue represents an overwhelming economic burden on the Unites States (USA) [2]. Farm animals have been attributed as one of the major reservoirs associated with transmission of various pathogens with poultry being the main animal source for several enteric bacterial pathogens, particularly various serovars of *Salmonella enterica* (SE) [3]. Restrictions on the sub-therapeutic use of antibiotics has also contributed an increase in bacterial load found in animal food products [4]. In addition to this, there is a growing trend of multiple antibiotic-resistant SE, with serovars such as *Salmonella enterica* serovar Typhimurium (ST) being of specific concern because of its sharply growing multi-antibiotic-resistant trend as well as its invasive capabilities regarding human intestinal epithelial cells and ubiquitous presence in multiple animal hosts, foods, and environments [5,6]. Though antibiotic stewardship has reduced some of the presence of antibiotics in animal products and the environment, these measures potentially expose consumers to food products that contain higher loads of pathogenic foodborne bacteria naturally found as part of animals’ normal microbial flora [7]. Despite these efforts, antibiotic-resistant bacteria have not been eliminated from these scenarios since antibiotic-resistance genes endure in the environment and within animal hosts, which serve as reservoirs for genes and other bacteria that can horizontally transfer and acquire these genes, even in the absence of previous antibiotic use [8]. Within this scenario, despite the abundance of antibiotics that are available for preventing and treating bacterial diseases such as salmonellosis, the increasing panorama of bacterial resistance to clinically relevant antibiotics has greatly limited the use case for these compounds [9]. Previous research has suggested that reducing the bacterial load of an animal at the pre-harvest level can lead to a decrease in pathogens present in the subsequent food product, which translates to a lower incidence of foodborne infection, in addition to a lower incidence of zoonosis, meriting the search for novel antimicrobial compounds that do not contribute to bacterial-resistance patterns [10].

ST is commonly found in the gastrointestinal (GI) system of poultry as part of its normal microbial flora and colonizes and thrives in the jejunum, ileum, and cecum without causing disease, while the chicks routinely shed the bacteria, releasing it in feces [11]. The cecum is one of the organs with the greatest abundance of bacteria, both in terms of numerical quantities and taxonomic diversity, consisting of a complex microbiome that includes bacteria that are commensal to the animal and non-pathogenic to humans, but also contains many pathogens including ST [12]. Therefore, cecum is an important organ of chickens as a source of poultry-borne enteric pathogens, and it can be targeted to control colonization of poultry-borne human pathogens. In addition, the bacterial dynamics that take place in the cecum must also be taken into account as these also have important implications for the host’s overall health [13].

Further, recent studies have revealed the importance of maintaining a diverse and stable gut microbiota for promoting and maintaining host health, and in the case of poultry, the cecal microbiome plays a major role in this process [12]. Other studies have proposed influencing the gut microbiome through the introduction of prebiotics and probiotics as a strategy for improving poultry health, welfare, and production as well as reducing the prevalence and colonization of pathogenic bacteria in their GI tracts, which would result in safer food products as well as less cross-contamination [14,15,16]. These specifically demonstrated the efficacy of plant extracts as both antimicrobial agents that could reduce the prevalence of common foodborne pathogens in an in vivo chicken model while also being capable of stimulating animal growth and promoting the microbial diversity within the guts of the animals that were given these products. Though these methods are promising and could be used to replace the use of conventional antibiotics in animal production, they require more study directed at understanding their mechanisms of action and how they can be implemented in a more targeted manner.

Recent findings indicate that plant extracts are promising candidates for discovering novel antimicrobials that contain a variety of diverse polyphenolic compounds [17]. Plant-derived flavonoids, tannins, and phenolic acids can be easily extracted and administered orally with little or no detriment to the animal and have proven benefits to them and their microflora [18,19]. Though there are many types of phenolic acids, each with their own unique chemical makeup and bioactive potential, many of them have been previously reported to have antimicrobial and anti-virulence properties against ST in vitro [20]. However, the effectiveness of pure phenolic acids on targeted pathogens in a complex biological system such as the cecum have not been studied well yet.

In this study, we aim to evaluate the effectiveness of gallic acid (GA), protocatechuic acid (PA), and vanillic acid (VA) in a simulated chicken-gut environment, specifically one containing cecal fluids collected from chickens, which contain the initial local bacterial communities commonly found in this animal as well as the surrounding matrix containing the nutrients, macromolecules, and compounds that form part of the contents in this part of the gut. This provides an environment that accounts for crucial interactions that could affect the efficacy and potential of pure, individual phenolic acids as future methods of microbial control while also serving as a model that can help elucidate more information with regards to the mechanism of action behind plant extracts. This study also seeks to evaluate gaps in compound potency associated to changes in medium composition used for testing antimicrobial compounds and environments in which these compounds are meant to be used. To address this, compound stability and potency was first evaluated within a wide pH range in nutrient-rich media. Effects on the growth pattern of ST in cecal fluid were also evaluated during treatment with phenolic acids. In addition to evaluating the effects of phenolic acids on ST, 16S-rRNA gene sequencing was used to evaluate changes in the microbial composition of the pre-exiting microbiota of the cecal fluid samples and later analyzed to determine changes in species diversity.

## 2. Materials and Methods

### 2.1. Bacterial Strain and Growth Conditions

*Salmonella enterica* serovar Typhimurium (ATCC 14028) (ST) was used for this study. Luria-Bertani (LB) (Becton, Dickinson and Co., Franklin Lakes, NJ, USA) agar and broth were used as the medium on which ST was grown, and it was incubated at 37 °C under aerobic conditions (Thermo Fisher Scientific Inc., Waltham, MA, USA). XLD (Hardy diagnostics, Santa Maria, CA, USA) and SS (Becton, Dickinson and Co., Franklin Lakes, NJ, USA) agar were used as differential media that allowed for the identification and differentiation of ST from other gram-negative coliforms.

### 2.2. Compounds and Stock Solution Preparation

The phenolic acid compounds that were assessed in this study were purchased in powder form from commercial vendors. Stock solutions were prepared for gallic acid (GA) (Acros Organics, Waltham, MA, USA) and protocatechuic acid (PA) (Sigma-Aldrich, Burlington, MA, USA) by dissolving it in sterile deionized water, while vanillic acid (VA) (Alfa Aesar, Ward Hill, MA, USA) was prepared by dissolving it in 30% ethanol (Pharmco-Aaper, Brookfield, CT, USA). Aqueous solutions of sodium hydroxide (NaOH) and hydrochloric acid (HCl) were both prepared to a molarity of 10 M and used for adjusting pH values of other solutions in this study. All compounds were prepared to contain a concentration of 10 mg/mL. Phosphate-buffered saline (PBS) was prepared to a pH of 7.2.

### 2.3. Phenolic Acid Antimicrobial Potential at Alternate pH Range

Antimicrobial potentials of phenolic acids against ST were evaluated at increasing concentrations of each compound within a pH ranging from 3 to 10 following previously described methods with certain modifications [21]. Briefly, LB broth samples were independently supplemented with increasing concentrations of GA, PA, and VA ranging from 0.07813 to 4.5 mg/mL, while aliquots of each concentration were concurrently adjusted to a specific pH value ranging from 3 to 10 using either HCl or NaOH, depending on the desired pH value of the solution being prepared. These solutions were inoculated with ST fixed to an optical density (OD_600_) of 0.1 in PBS (10^8^ CFU/mL) that was further diluted to achieve a final bacterial load of 10^4^ CFU/mL in the final volume. These samples were incubated aerobically for 24 h at 37 °C and screened for visible growth, which was confirmed through plating in LB agar. Growth in LB agar was interpreted as a sign of inactivation of the phenolic acid either by changes in pH and/or changes in minimum inhibitory concentration (MIC).

### 2.4. Simulated Chicken Cecum Model Design

Previous research on the antimicrobial potential of these phenolic acids against ST (ATCC 14028) determined the lethal concentration (elimination of bacteria to the point of no detectable growth) to be 4.5 mg/mL for GA, 2 mg/mL for PA, and 1.5 mg/mL for VA with minimal change in susceptibility over various passages [20]. A simulated chicken cecal environment was designed in order to study the effects that the phenolic acids GA, PA, and VA have on a complex environment with a diverse bacterial community such as the chicken gut while also evaluating differences in the effectiveness of these compounds when it comes to inhibiting the growth of ST. This model was designed as has been described in the past by previous research groups with some modifications [22]. Briefly, to attain the complex microbial composition of the gut, as well as the environmental matrix that they inhabit, cecal fluid samples were collected from two groups of previously sacrificed chickens (*Gallus gallus domesticus*), one being comprised of 3 21-day-old birds and the other of 4 28-day-old birds (IACUC, protocol number R-FEB-20-04). The two groups with their respective sets of birds served as duplicates, while cecal contents from each bird within a group were combined to account for variances between birds. These were dissected to remove the cecum, which was later emptied of the cecal fluid and suspended in PBS immediately after the initial collection. Samples belonging to their respective collection group were pooled together and homogenized through vortexing to ensure equal distribution of cecal contents across multiple samples as well as initial microbial composition. Groups consisted of an untreated control group and three treatment groups individually supplemented with previous experimentally determined lethal doses of each corresponding compound, namely GA (4.5 mg/mL), PA (2 mg/mL), and VA (2 mg/mL) [20]. All samples were inoculated with a bacterial suspension of ST previously prepared by using ST grown overnight in LB agar at 37 °C, and later fixed to an OD_600_ of 0.1 in PBS. The final concentration of bacteria in each sample solution was 10^4^ CFU/mL. After inoculation, samples were incubated for 24 h and 48 h at 37 °C under aerobic conditions, and sample aliquots of 1 mL were taken at 0 h, 24 h, and 48 h, part of which was used for determining ST growth and the rest were stored for later total DNA extraction. The pH was measured across all timepoints for all treatment types and controls using a pH probe to identify any deviations from the initial pH.

### 2.5. Quantification of ST Growth after Treatment with Phenolic Acids

The growth of ST was quantified in all samples (Control, GA, PA, and VA) from both duplicate collection groups at the three predetermined timepoints (0 h, 24 h, and 48 h). This was performed through a microdilution assay, as has been described before [23], with some modifications. Aliquot from each sample were taken at the pre-determined timepoints and diluted using PBS before plating in XLD and SS. The media selected allowed for the differentiation of coliforms in samples containing a mixed culture of bacteria with only bacteria that developed black colonies being considered for quantification and counted as positive for ST [24]. CFU/mL values were calculated for each sample and later presented in Logs CFU/mL.

### 2.6. 16S-rRNA Microbiome Analysis

The microbial composition of each sample was evaluated through the use of 16S-rRNA gene sequencing as has been described before [14]. Briefly, total DNA was extracted from all samples using a QIAamp Fast DNA Stool Kit (QIAGEN, Germantown, MD, USA) according to the manufacturer guidelines. The extracted DNA was quantified using a NanoVue NanoDrop (GE Healthcare, Chicago, IL, USA) to validate the extraction yield and later normalize samples to a fixed concentration. Taxonomic analysis was performed with amplification of the variable v3 and v4 regions of 16S-rRNA, allowing for phylogenetic classification of the bacteria in the sample through next-generation sequencing and further diversity analysis. Briefly, Nexter DNA Library Preparation Kit (Illumina, San Diego, CA, USA) was used to prepare samples for sequencing. KAPA HiFi HotStart ReadyMix, primer amplicons, and DNA samples were combined, and a PCR reaction was performed with the following protocol: 95 °C for 3 min; 25 cycles of 95 °C for 30 s, 55 °C for 30 s, and 72 °C for 30 s; and 72 °C for 5 min. Product cleanup for eliminating free primers and primer dimer species was performed using AMPure XP beads. Index PCR was later performed to generate a library using a Nextera Index Kit (Illumina, San Diego, CA, USA), following the manufacturer guidelines. The PCR reaction was carried out as follows: 95 °C for 3 min; 8 cycles of 95 °C for 30 s, 55 °C for 30 s, and 72 °C for 30 s; and 72 °C for 5 min. Library products were cleaned for a second tie using the AMPure XP beads. Library concentrations were quantified and normalized in order to prepare an equimolar pool of DNA amplicons, which was later combined with the PhiX reference control provided by the manufacturer. After heat denaturation (96 °C for 2 min), the sample pool was loaded, and pair-end: 2 × 300 bp sequencing was performed using a MiSeq v3 600 Cycle Kit (Illumina, San Diego, CA, USA) in the Illumina MiSeq System (Illumina, San Diego, CA, USA).

### 2.7. Analysis of Taxonomic Abundance

A total of 11,903,602 quality-filtered reads were used for taxonomic analysis and profiling of each sample. Sequence analysis was performed using the MiSeq Reporter-BaseSpace workflow tool for FASTQ, which uses the Greengenes Database. Further analysis involved the taxonomic quantification and classification of the bacterial profile of each sample at the phylum and genus levels [25]. The mean richness and diversity at the species level were analyzed in each sample by determining alpha (α) diversity, which was determined by using the Shannon Index as has been established before [26]. Evenness at the species level for each sample was also determined by calculating the Inverse Simpson Index [27]. Similarity at the species level and species overlap between treated samples when compared to control were also analyzed by determining beta (β) diversity, which was calculated using Sorenson’s Coefficient [28]. The number of operational taxonomic units (OUT) in each sample was calculated for each sample and compared between respective timepoints using a Venn diagram for further comparison and visualization, which was generated using the web-based InteractiVenn tool [29].

$$\mathrm{Shannon}\;\mathrm{Index}\;(H):\;\;Proportion\;\left(p\right)=\frac{Individuals\;of\;one\;species\;\left(n\right)}{Total\;number\;of\;individuals\;\left(N\right)}$$

$$H=-\overset{s}{\underset{i=1}{\sum }}p_{i}\ln p_{i}$$

$$\text{Inverse Simpson Index}\;(D):\;D=1/(\overset{s}{\underset{i=1}{\sum }}p_{i}^{2})$$

$$\text{Sorenson’s Coefficient}\;(CC):\;Number\;of\;species\;in\;common=C\;Total\;number\;of\;species\;found\;in\;community=S_{n}\;CC=\frac{2C}{S1+S2}$$

### 2.8. Statistical Analysis

Student’s *t*-test and ANOVA were used to determine statistically significant (*p* < 0.05) changes between individually treated groups with the untreated control.

## 3. Results

### 3.1. Antimicrobial Potency of GA, PA, and VA at Various pH Ranges

The antimicrobial potency of increasing concentrations of the three phenolic acids in LB broth were evaluated by adjusting the pH of each individual solution to a value ranging from pH 4 to pH 9 (Table 1). ST was used to inoculate each solution containing increasing concentrations of either GA, PA, or VA (0.0156–4.5 mg/mL) in addition to a control containing only LB broth, all of which were fixed to a specific pH value. After incubation of ST with each unique mixture for a 24 h period, the samples were screened for signs of visible growth and confirmed via plating in LB agar. Changes in pH altered the inhibitory efficacy of each compound significantly, leading to either their inactivation or increasing their potency, requiring less concentration of each of the compound to inhibit ST growth to undetectable levels. As a control, an initial testing of tolerance to a wide range of pH values was assessed only in LB broth, finding ST to be unable to grow below a pH of 4, demonstrating limited growth above pH 9. Considering the antimicrobial pressure these levels exert on the bacteria without the addition of any of the compounds being evaluated in this study led to the selection of the current pH ranges. Further changes in antimicrobial activity were observed in GA, which retained inhibitory potential against ST across the widest range of pH values, only showing loss of function at pH 4, up until the concentrations of the compound fell below 1 mg/mL, at which point there was loss of function at pH 5 as well. Potency of GA saw an improvement between the ranges of pH 6 to pH 9, lowering the lethal dose of the compound to 0.125 mg/mL and to 0.0625 mg/mL at pH 8 and above. Efficacy of GA was lost at concentrations below 0.0313 mg/mL in all pH levels. PA showed inactivation between pH levels of 5 and 7 at 2 mg/mL, but also demonstrated an improvement in potency at pH 8 similar to GA, as the lethal dose of PA required for inhibition of ST growth was lowered to 0.125 mg/mL, while retaining efficacy up to 0.0313 mg/mL at pH 9. VA showed the lowest tolerance in changes in pH with inactivation between pH 5 and pH 6, even at higher concentrations. At 2 mg/mL of VA, there was inactivation between the ranges from pH 5 to pH 9, evident in the loss of antimicrobial activity. At pH 4, VA showed an improvement in antimicrobial effect, inhibiting all detectable growth of ST at a lower concentration of 0.5 mg/mL (Table 1).

### 3.2. Quantification of ST Growth in Chicken Cecum Fluids in the Presence or Absence of GA, PA, or VA

**Figure 1:**
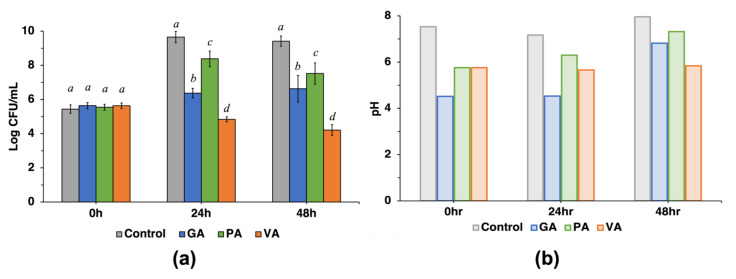
Antimicrobial effect of gallic acid (GA), protocatechuic acid (PA), and vanillic acid (VA) on Salmonella enterica serovar Typhimurium (ST) in cecum fluid conditions measured in Log CFU/mL at the 0 h, 24 h, and 48 h timepoints (**a**), while pH was measured at the same timepoints at which sample aliquots were taken (**b**). Statistically significant (*p* < 0.05) values between treatments when compared to the untreated control at the respective timepoints are denoted with letters *a–d*.

Growth of ST in the simulated cecal environment was evaluated by collecting cecum fluids from each time point and quantifying the bacterial load (CFU/mL) using plate microdilution assay. The bacterial count for each sample was calculated and converted to Log CFU/mL (Figure 1). The moment of inoculation with ST in the extracted cecal fluid was used as the starting point for the time scale at 0 h. At the initial timepoint (0 h), there was no significant difference between the control and the treatments (Figure 1a). After 24 h, ST population grew to 9.65 Log CFU/mL in the untreated control with CFU/mL values being significantly (*p* < 0.05) lower in treated cecum fluids at subsequent timepoints. After 24 h, ST in samples treated with GA and PA increased slightly to 6.37 and 8.38 Log CFU/mL, increasing form the initial inocula at 0 h, but remaining lower than the untreated control. In the same assay and timepoint, VA decreased ST from its initial concentration to 4.84 Log CFU/mL. After 48 h, ST concentration in the untreated control showed a slight decrease to 9.41 Log CFU/mL, while values for the treated sample remained lower. Despite remaining lower than the untreated control, growth of ST was increased slightly in cecum fluids to 6.63 Log CFU/mL and 7.52 Log CFU/mL in GA- and PA-treated samples at the 48 h timepoint, respectively, whereas in the same assay, the cecum fluids treated with VA, growth of ST was reduced to 4.21 Log CFU/mL. The pH was measured at each timepoint for the non-treated control and treated samples (Figure 1b), finding the initial pH of the untreated control to be pH 7.53, which decreased to pH 7.17 after 24 h and later increased to 7.96 after 48 h. Supplementation with phenolic acids provoked a significant change in initial pH as the GA treatment group started at pH 4.52, but gradually increased to pH 4.53 and pH 6.82 after 24 h and 48 h, respectively. Initial pH in the PA group was slightly higher than GA with a pH of 5.76, but also increased gradually to pH 6.3 and pH 7.32 after 24 h and 48 h, respectively. VA was initially the same pH as PA as it also started at a pH of 5.76; however, fluctuations over time were less drastic as it decreased to pH 5.66 at 24 h and later to pH 5.84 at 48 h, showing less variation than the other treatment groups.

### 3.3. Changes in Microbial Makeup at Major Phyla and Genus Levels Due to Treatments

**Figure 2:**
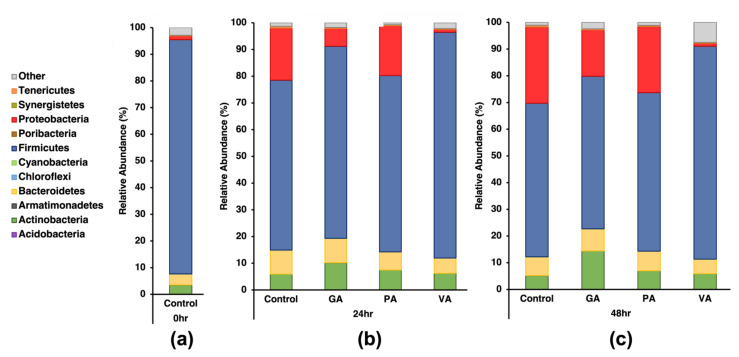
Relative percentage of abundance of microbes at the phylum level for the top 12 phyla in 16S-rRNA gene sequencing data at 0 h (**a**), 24 h (**b**), and 48 h (**c**) for an untreated control and groups individually treated with either gallic acid (GA), protocatechuic acid (PA) and vanillic acid (VA).

**Figure 3:**
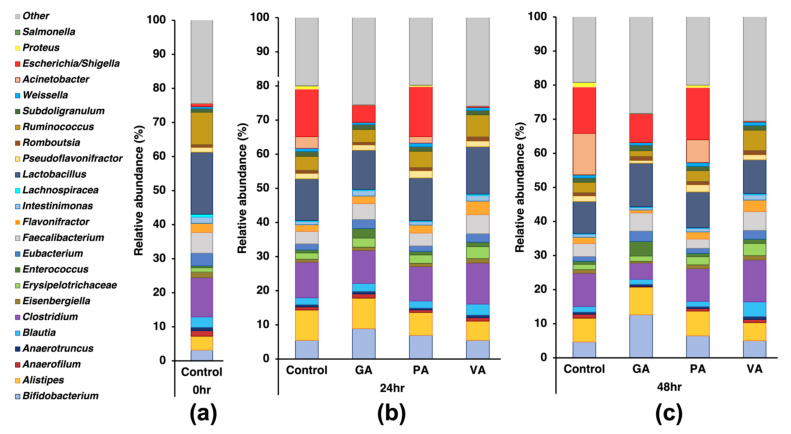
Relative percentage of abundance of microbes at the genus level for the top 25 genera in 16S-rRNA gene sequencing data at 0 h (**a**), 24 h (**b**), and 48 h (**c**) for an untreated control and groups individually treated with either gallic acid (GA), protocatechuic acid (PA) and vanillic acid (VA).

Changes in bacterial population distribution as a result of treatment with phenolic acids were studied using 16S-rRNA sequencing, which was later analyzed to uncover the taxonomical composition in each sample at both the phylum and genus levels (Figure 2 and Figure 3, respectively). The experiments were performed with a technical duplicate for each biological replicate, and the results shown are the averages of the independent values obtained from each run. Relative bacterial abundance was determined from the percentage (%) of total reads classified to the respective taxonomic level, which at the phylum level was an average rate of 97.80% and at the genus level was an average rate of 86.50%. Changes in the bacterial populations in the samples treated with the respective phenolic acids were compared to the untreated control at the same timepoints. Reads from the control at 0 h revealed the four major phyla to be Firmicutes (87.76%), Bacteroidetes (4.14%), Actinobacteria (3.44%), and Proteobacteria (1.38%). At the 24 h timepoint for the untreated control, the top four major phyla remained the same as at 0 h; however, the relative abundance of Firmicutes decreased (63.56%), while the relative abundance increased for Bacteroidetes (8.98%), Actinobacteria (5.91%), and Proteobacteria (19.51%). Samples treated with GA at the same timepoint showed a statistically significant (*p* < 0.05) increase in Firmicutes (71.86%), Actinobacteria (10.72%), and in the less-abundant Cyanobacteria (0.05%), while there was a significant decrease in Proteobacteria (6.65%). Samples treated with PA showed a slight numerical increase in Firmicutes. A significant change (*p* < 0.05) was seen in a decrease in Bacteroidetes (6.76%) and the less-abundant Synergistetes (0.02%). Samples treated with VA showed a significant (*p* < 0.05) increase in the abundance of Firmicutes (84.45%) and Cyanobacteria (0.03%), while there was a significant reduction in Proteobacteria (1.08%) and Bacteroidetes (5.70%).

At the 48 h timepoint for the untreated control, the top four major phyla remained the same as at 24 h with a numerical decrease in the relative abundance of Firmicutes (57.50%) and Actinobacteria (5.13%), while there was an increase in Proteobacteria (28.59%) and Bacteroidetes (7.02%) with the latter being significant (*p* < 0.05). Samples treated with GA only saw a statistically significant (*p* < 0.05) change in the increase in Actinobacteria (14.40%) and the less abundant Cyanobacteria (0.04%); however there was a numerical decrease in Proteobacteria (17.38%) and increase in Bacteroidetes (8.21%). Samples treated with PA only showed numerical variations when comparing the top phyla with the untreated control, with a decrease in the less abundant Tenericutes (0.32%) being significant (*p* < 0.05). Samples treated with VA showed a statistically significant (*p* < 0.05) increase in the abundance of Firmicutes (79.69%), while there was a decrease in Proteobacteria (1.12%) and Tenericutes (0.19%).

Taxonomic classification at the genus level was performed by calculating the percentage of relative abundance for the top 45 bacterial genera discovered in each sample, while genera that fell below these values were added and placed under *Other*. The ten most-abundant genera at timepoint 0 h were *Lactobacillus* (18.22%), *Clostridium* (11.54%), *Ruminococcus* (9.50%), *Faecalibacter* (6.07%), *Alistipes* (4.08%), *Eubacterium* (3.74%), *Bifidobacterium* (3.17%), *Blautia* (3.07%), *Flavonifractor* (2.69%), and *Intestinimonas* (1.82%), while *Salmonella* (0.004%) was below these values. At the 24 h timepoint, the ten most-abundant genera were *Escherichia/Shigella* (13.91%), *Lactobacillus* (12.19%), *Clostridium* (10.45%), *Alistipes* (8.91%), *Bifidobacterium* (5.45%), *Ruminococcus* (4.10%), *Faecalibacterium* (3.66%), *Acinetobacter* (3.46%), *Blautia* (2.05%), and *Flavonifractor* (1.98%) with *Salmonella* (0.059%) increasing over this time. Of these, when comparing to the initial 0 h timepoint control, the increase in *Escherichia/Shigella* and *Alistipes*, as well as the decrease in *Eubacterium*, were statistically significant (*p* < 0.05). Samples treated with GA showed a significant increase in *Bifidobacterium* (8.90%) and *Faecalibacter* (4.64%), though there was a slight numerical decrease in *Lactobacillus* (11.50%), and there was also a decrease in *Escherichia/Shigella* (5.03%), *Clostridium* (9.64%), and *Ruminococcus* (3.77%) as well as a slight increase in *Salmonella* (0.079%). Samples treated with PA had a similar abundance of the top genus compared to the untreated control with *Alistipes* (6.71%) being significantly (*p* < 0.05) decreased, while *Salmonella* (0.043%) was lower. Samples treated with VA had a similar abundance in the top ten genera, demonstrating a numerical increase in *Lactobacillus* (13.90%) and *Clostridium* (12.05%) with a significant (*p* < 0.05) change being observed in the abundance of *Escherichia/Shigella* (0.37%) and *Alistipes* (5.66%) along with an increase in the abundance of *Ruminococcus* (6.49%), while *Salmonella* (0.0025%) experienced numerical reduction.

At the 48 h of timepoint for the untreated control, the top ten bacterial genera were *Escherichia/Shigella* (13.57%), *Acinetobacter* (12.19%), *Clostridium* (9.77%), *Lactobacillus* (9.38%), *Alistipes* (6.96%), *Bifidobacterium* (4.68%), *Faecalibacterium* (3.85%), *Ruminococcus* (3.09%), *Flavonifractor* (1.79%), and *Pseudoflavonifractor* (1.66%) with *Salmonella* below these values (0.051%). When compared to the 24 h timepoint for the untreated control, a statistically significant (*p* < 0.05) change was seen in an increased abundance of *Acinetobacter* and a decrease in *Alistipes*. Samples from the same timepoints treated with GA demonstrated a change in the distribution of the previously reported top ten genera as there was a numerical increase in *Lactobacillus* (12.82%), *Alistipes* (8.13%), *Klebsiella* (7.28%), and *Salmonella* (0.12%) with statistical significance (*p* < 0.05) found in *Bifidobacterium* (12.62%), *Enterococcus* (4.29%), and *Eubacterium* (3.01%). On the other hand, there was a numerical reduction in *Escherichia/Shigella* (8.44%), *Clostridium* (9.77%), and *Ruminococcus* (1.73%) with significance being found in *Clostridium* (4.74%), *Acinetobacter* (0.02%), and *Pseudoflavonifractor* (0.81%). Samples treated with PA showed a numerical increase in *Escherichia/Shigella* (15.29%), *Lactobacillus* (10.53%), and *Bifidobacterium* (6.45%) with *Klebsiella* (0.75%) being statistically significant (*p* < 0.05), while a decrease was observed in *Faecalibacterium* (2.71%) and *Salmonella* (0.038%) with *Acinetobacter* (6.70%) being statistically significant (*p* < 0.05). Samples treated with VA demonstrated a numerical increase in *Clostridium* (12.32%), *Ruminococcus* (6.01%), and *Eubacterium* (2.61%) with *Flavonifractor* (3.40%) being statistically significant (*p <* 0.05), while there was a numerical decrease in *Salmonella* (0.002%) with *Escherichia/Shigella* (0.21%), *Acinetobacter* (0.055%), and *Klebsiella* (0.018%) being significant (*p* < 0.05).

### 3.4. Changes in Bacterial Composition Assessed through Alpha and Beta Diversity Indexes at Species Level Due to Treatments

**Figure 4:**
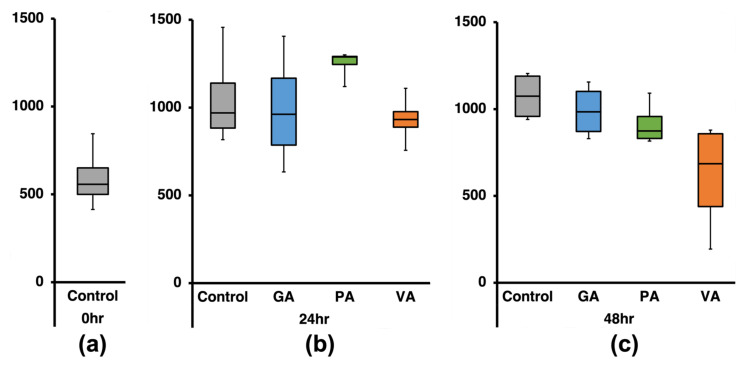
Number of identified species in 16S-rRNA gene sequencing data at 0 h (**a**), 24 h (**b**), and 48 h (**c**) for an untreated control and groups individually treated with either gallic acid (GA), protocatechuic acid (PA) and vanillic acid (VA).

**Figure 5:**
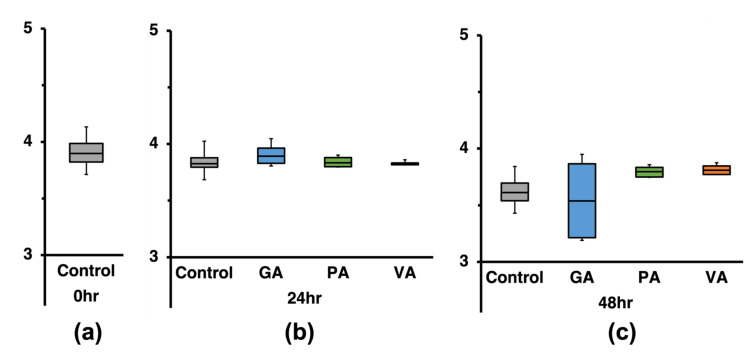
Alpha diversity analysis at the species level using the Shannon Index from 16S-rRNA gene sequencing data at 0 h (**a**), 24 h (**b**), and 48 h (**c**) for determining diversity for an untreated control and groups individually treated with either gallic acid (GA), protocatechuic acid (PA) and vanillic acid (VA).

The taxonomic distribution of bacteria at the species level was analyzed based on the percentage (%) of total reads classified at this taxonomic level, which was on average 50.96% from the total classified reads. The species number was quantified (Figure 4), while further analyses on species distributions and diversity were performed using calculations for alpha distributions (Shannon Index and Inverse Simpson Index) in addition to comparing distribution between classified bacterial communities within samples through beta distribution (Sorrenson’s Coefficient) (Figure 5). At the 0 h timepoint, there was an average of 593 species identified (Figure 4a), while at the 24 h (Figure 4b) timepoint there was a statistically significant (*p* < 0.05) increase in the average number of species identified (1053). When comparing the number of species in the samples treated with phenolic acids to that of the untreated control at the same timepoint, there was a slight numerical decrease in GA and VA (991 and 932, respectively), while in PA there was an increase (1249). At 48 h (Figure 4c), there was a slight increase in the species number (1073), but it was not statistically different from the 24 h timepoint. When comparing species among samples at 48 h, samples treated with phenolic acids demonstrated lower numbers of species in the cases of GA (988), PA (914), and VA (611) compared to the untreated control.

**Figure 6:**
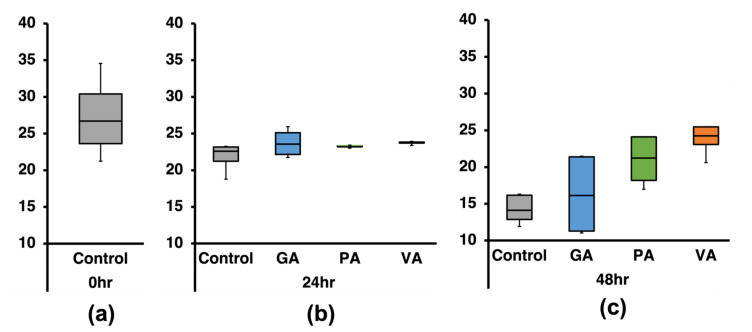
Alpha diversity analysis at the species level using Inverse Simpson Index from 16S-rRNA gene sequencing data at 0 h (**a**), 24 h (**b**), and 48 h (**c**) for determining evenness for an untreated control and groups individually treated with either gallic acid (GA), protocatechuic acid (PA) and vanillic acid (VA).

The Shannon Index was calculated as an indicator of alpha diversity for each sample in order to determine microbial diversity within each one (Figure 5). The untreated control at the 0 h (Figure 5a) timepoint had a value of 3.91 with a slight decrease to 3.85 and later 3.62 at 24 h and 48 h, respectively. Comparatively, at 24 h (Figure 5b), samples that were treated showed a slight numerical decrease compared to the untreated control, as seen in the slightly lower values for GA (3.90), PA (3.84), and VA (3.82). At 48 h (Figure 5c), values were lower than their 24 h counterparts, as seen in values for GA (3.54), PA (3.79), and VA (3.81), despite the latter two being higher than the untreated control at the same timepoint. The Inverse Simpson Index (Figure 6) was used as another indicator of alpha diversity in terms of evenness, which showed the untreated control at 0 h to be 27.30 (Figure 6a) and to also decrease slightly to 21.80 and then significantly to 14.92 (*p* < 0.05) over 24 h and 48 h periods, respectively. Further, at 24 h (Figure 6b), values for the samples treated with GA, PA, and VA were 23.70, 23.24, and 23.73, respectively, which was slightly higher than the untreated control, but was still lower than the 0 h sample. At 48 h (Figure 6c), values for the samples treated with GA and PA decreased further to 16.54 and 21.08, respectively, while VA significantly increased to 24.29 (*p* < 0.05).

**Figure 7:**
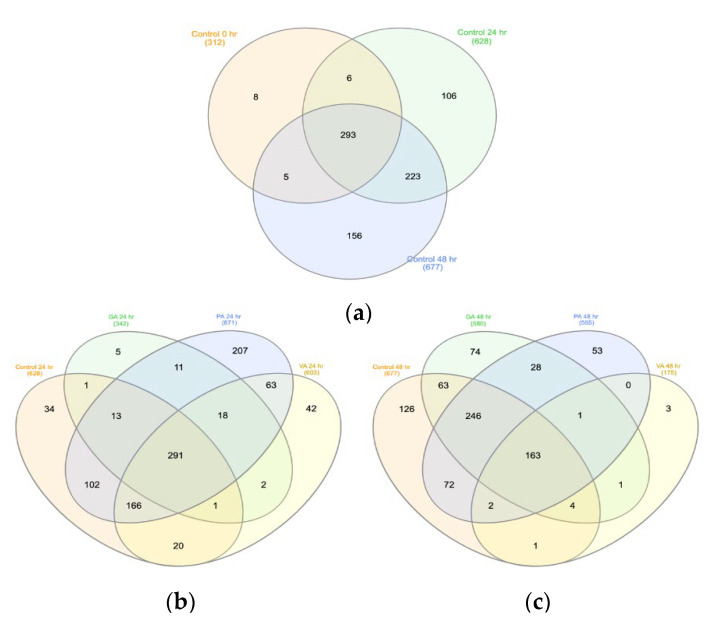
Multivariate Venn diagram demonstrating species commonness between sample OTUs by comparing controls at different timepoints (**a**) as well as the control, gallic acid (GA), protocatechuic acid (PA), and vanillic acid (VA) treatment groups at the 24 h timepoint (**b**) and control, GA, PA, and VA treatment groups at the 48 h timepoint (**c**).

**Figure 8:**
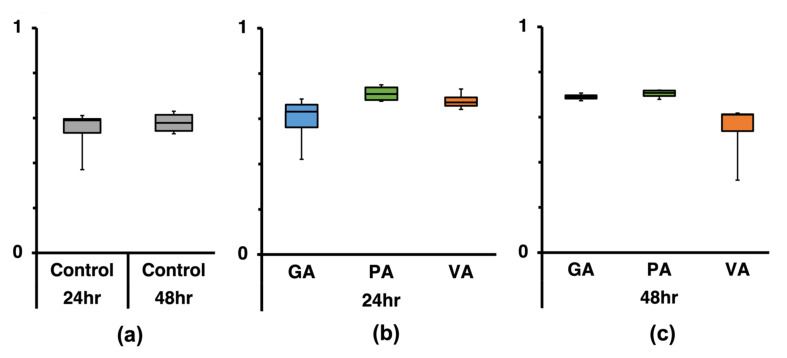
Beta diversity analysis at the species level using Sorenson’s Coefficient from 16S-rRNA gene sequencing data calculated by assessing overlap between the population of species found in untreated controls at 0 h with those at 24 h and 48 h (**a**) as well as assessing overlap between gallic acid (GA), protocatechuic acid (PA) and vanillic acid (VA) with their respective untreated control within the same timepoints, namely 24 h (**b**) and 48 h (**c**).

Further analysis of the species number required finding the common bacteria among the replicates of each group, which allowed for the comparison of OUT between the control and treatment groups at a given timepoint using a Venn diagram for each timepoint (Figure 7). When considering only the number of common species found across all replicates of each group, there was a total of 312 common species identified in the control at the 0 h timepoint (Figure 7a), while these numbers increased to 628 and 677 at the 24 h (Figure 7b) and 48 h (Figure 7c) timepoints. After 24 h of treatment, the number of common species within the replicates of each group was 342, 871, and 603 for GA, PA, and VA, respectively. After 48 h of treatment, the common species within replicates of each treatment group increased to 580 for GA but reduced to 565 and 175 for PA and VA, respectively. Using the three-way Venn diagram tool allowed us to evaluate common species amounts for the control and treatment groups at each timepoint. There were 293 core species detected between all three control timepoints with the 24 h and 48 h timepoints sharing 223 common species, which was higher compared to the 0 h timepoint. When comparing common species between groups within the 24 h timepoint using a four-way Venn diagram, there was a total of 291 core species detected between all treatment groups and control. Control and PA had the most similarity as they shared an additional 102 species in common. At the 48 h timepoint, there was a total of 163 core species across all groups with Control and PA having the most in common as they share an additional 72 species in common. The microbial distribution at the species level between bacterial communities in each sample was further analyzed by calculating and comparing the beta distribution between respective treated samples and their corresponding untreated control. Beta distribution between communities was determined by calculating the Sorenson’s Coefficient (Figure 8). The coefficients calculated from the comparison between untreated samples at 0 h with those from the 24 h and 48 h timepoints (Figure 8a) were 0.59 and 0.61, revealing similarity; however, when comparing the bacterial communities between the 24 h and 48 h timepoints, the coefficient value increases to 0.71, suggesting that these possess more species overlap. When comparing the similarities between untreated and treated bacterial communities at the 24 h timepoint (Figure 8b), the values for GA, PA, and VA were 0.59, 0.711, and 0.68. When comparing the similarities between untreated and treated bacterial communities at the 48 h timepoint (Figure 8c), the values for GA, PA, and VA were 0.69, 0.70, and 0.54.

## 4. Discussion

The search for novel antimicrobials, which can be used in farm animal production, remains an important endeavor for the future of food production and safety of products. However, recent uptrends in antibiotic resistance, particularly in enteric bacterial pathogens and current knowledge regarding the importance of maintaining balanced microbiomes require careful consideration. Additionally, novel antimicrobials should not have negative effects on the communities of beneficial and commensal bacteria in or on the host, and they should inhibit targeted pathogens. Further, these compounds should also aid in making food products safer while also meeting demand, keeping animals healthy, and preventing cases of zoonosis. Direct elimination of bacterial pathogens through the implementation of antimicrobials remains one of the main strategies for improving food safety; however, influencing the microbiome through bioactive compounds has been demonstrated to also be a promising approach to achieve the aforementioned goals in the industry [30]. Maintaining a diverse gut microbiome has been established as a hallmark of good health as it contributes to better bioavailability of nutrients and weight gain in chicken models [14]. A balanced gut microbiome also helps in the prevention of pathogenic bacterial colonization of the GI tract through competitive exclusion and growth inhibition by producing other bioactive antimicrobial metabolites [31]. The compounds used in this study (GA, PA, and VA) had been previously tested in vitro against ST, in which they demonstrated antimicrobial potential [20]; however, the current study expands on the potential for these compounds to be used in scenarios closer to those that would be encountered inside of the chicken GI tract and evaluates their effect on the normal microbial flora that is commonly found there.

Controlling pH with weak acid has been used as a method for increasing their antimicrobial potency in food products as they are often used as preservatives [32]. In the case of GA, PA, and VA, pH was found to have a significant effect on the antimicrobial potential of each compound. GA not only retained its antimicrobial potential across a wider range of pH and concentrations, but also saw an improvement in activity at certain values, whereas PA lost activity closer to neutrality, while retaining and improving at the ends of the range tested, whereas VA lost much of its antimicrobial potential at most of the pH values tested, once tested at concentrations below the previously determined MIC. A similar antimicrobial pattern has been previously reported in other organic weak acids wherein researchers have reported an optimization in inhibition of bacterial growth based on the pH of a given compound at the time of the assay [21]. Explanations for this have been proposed to be related to the associated and/or disassociated state of organic acids at specific pH values, which influences their capability to freely diffuse through the bacterial envelope. This has been reported in both gram-positive and gram-negative bacteria but is especially relevant in the latter since their outer membranes are often viewed as resistance structures that are to be overcome in order for antimicrobial compounds to be effective in inhibiting their growth [33,34]. Lower pH values have been associated with more associated states in weak acids, which potentially makes some of them more effective at inhibiting bacterial growth. However, this correlation has been found to also be dependent on the ratio between the pH and pKa values of each individual compound as it has been found to play a significant role in the interactions that are possible with specific bacteria [33]. In this study, even at lower concentrations, higher pH values passed neutrality (pH 7) and consistently sustained higher antimicrobial activity, especially in GA and PA, though the former was effective up to a pH of 8 and concentration of 0.0625 mg/mL, and the latter was effective up to a pH of 9 and a concentration of 0.0313 mg/mL. Though the effects of phenolic acids could also be amplified by certain bacterial stressors associated with alkaline stress and enzyme inactivation at these higher ranges of pH, they require further investigation into the mechanics involved in weak acid activity when fixed to higher pH levels since these still fall within the commonly understood range that is permissible for ST growth [35].

When evaluating the antimicrobial potential of these phenolic acids against ST in the simulated cecal environment, there was a reduction in potency compared to previous reports. GA had the ability to limit the growth of ST over time compared to the untreated control. However, there was no complete elimination of the bacteria despite the use of the previously determined lethal dose of the compound. VA demonstrated the most significant reduction of ST by reducing more ST with each increasing timepoint while also being the only treatment to reduce the bacterial count to a value below the initial inoculation size. PA had the least effective outcome of the phenolic acids evaluated as it showed no significant reduction in bacterial growth over any of the timepoints, allowing for the proliferation of ST to reach levels similar to that of the untreated control. Though previous research demonstrated GA to be the least potent of the three compounds in vitro, in this study, it proved to be more effective than PA, which resembles it in structure. GA has an additional hydroxyl group (-OH) compared to PA, while VA has a methoxy group (-OCH_3_). The additional functional hydroxyl group could make GA more resistant to oxidation, especially in environments with varying pH ranges [36]. The difference in functional groups also contributes to the polarity of each compound, which influences the compound’s ability to freely diffuse across the bacterial membrane [37]. In addition to oxidation, commensal gram-positive microbes have been found to break down phenolic compounds and use them as carbon sources, which is a process that has been reported to either confer antimicrobial potential to the resulting products of these reactions or can lead to their inactivation as the microbes further break them down [38,39,40]. The pH at each timepoint could also have played a role in the potency of each compound at a given timepoint. While GA was able to remain effective at a wider range of pH values when altered manually, in the cecal fluid setting, it was initially at a pH of 4.52, while in vitro, pH 4 showed to lower the potency of the compound. At a pH of 5, GA regained efficacy. This could serve as an explanation for how GA was able to reduce some of the bacterial load at 24 h and maintain ST at similar levels over 48 h. On the other hand, PA, though requiring lower concentrations for antimicrobial potency in vitro, showed a loss of potency at a range between pH 5 and 6, which coincides with the results seen in the cecal fluid wherein pH levels between 0 h and 24 h fell within this range. This could have been a factor that contributed to the lower efficacy of PA in the cecal samples. The effects on pH for VA when in the cecal fluid were not as clear, since, despite it losing efficacy in vitro at a pH between 5 and 9, it was the most effective in reducing ST even though the pH of the simulated cecal environment did not drop lower than pH 5 during the treatment. This could be related to the different compound structure of VA, which makes it more resistant to oxidation and disassociation in natural environments but could also be related to the bacteria being more favored in vitro by a specific compound in LB broth that makes them more resistant to VA in these altered pH ranges [41]. These results highlight the importance of understanding the final pH at which a compound is intended to use, since it might influence the potency of the compound, especially in those as dynamic as phenolic acids, which might also favor those that are more resistant to these changes or more electrochemically stable.

Results from the 16S-rRNA gene sequencing and subsequent microbiome analysis revealed that Firmicutes, Bacteroidetes, Actinobacteria, and Proteobacteria were the most abundant phyla, which is consistent with the pre-established literature regarding the composition of chicken microbiomes in the cecum [42,43]. These phyla persisted as the most abundant across timepoints and treatments, though they did experience changes in the percentages of the abundance ratios within each group that were time and treatment dependent. Firmicutes, which mostly consist of gram-positive probiotic and commensal bacteria, were the most abundant in all sample groups, though they were gradually reduced over time in the untreated control, which had no antimicrobial pressure. Though Bacteroidetes and Actinobacteria increased in abundance over time in the untreated control, Proteobacteria, which mostly consist of gram-negative pathogenic bacteria, showed the most significant increase under the same conditions. When treated with GA and VA, Proteobacteria were less abundant than in untreated samples at the same timepoint while also preserving the number of Firmicutes, as seen in a Firmicute abundance more similar to that of the one found at the initial conditions of the cecal fluid before incubation. Of the three compounds tested, the one with the most significant reduction of Proteobacteria and promotion of Firmicutes was VA, followed by GA, with PA showing a slight numerical difference from the untreated control at the same timepoint.

Analysis at the genus level also showed that some of the most notable genera that were greatly represented within each phylum belonged to either Firmicutes, Proteobacteria, Bacteroidetes, or Actinobacteria. Within the phylum Firmicutes, the most common genera found were common probiotics such as *Lactobacillus* and *Ruminococcus*, except for *Clostridia*, which, depending on the species, can be either considered part of the normal flora or could be an opportunistic pathogen [44]. Within the phylum of Bacteroidetes, which normally mostly consists of gram-negative bacteria, many of which are known to colonize the GI tract of animals [45], the genus *Alistipes* was found to be the most abundant. This bacterium genus has been discovered recently, but recent data suggest that it is commonly found within the cecum of chickens and has been found to show performance- and growth-promoting activity associated with broiler chickens [46,47]. Within the Actinobacteria phylum, there are mostly gram-positive soil bacteria such as *Bifidobacterium*, a known probiotic bacterium that has been studied and implemented in chicken models, which exhibited an improvement in health parameters as well as demonstrated the potential to reduce the colonization of commonly known gram-negative foodborne pathogenic bacteria [48,49]. The Proteobacteria phylum is most associated with harboring a vast array of common gram-negative pathogenic bacteria, including those belonging to the genus of *Salmonella*. However, in this study, the most predominant genus within this phylum was *Escherichia*. This is a genus consisting of common enteric bacteria that can be commensal, but also have highly pathogenic and virulent serotypes that are common foodborne pathogen knowns for contaminating meat and produce that can lead to infection, causing diarrheal disease and other complications [50,51].

Changes in the relative abundance of the main genera within each group varied across time and treatment. The most notable changes were seen in *Lactobacillus*, which naturally deceased in every group over time; however, when treated with GA, PA, and VA, their relative abundance remained numerically similar or higher than the untreated control at a given timepoint, showing no detrimental effect of the compounds on this genus. Other gram-positive Firmicutes such as *Ruminococcus* naturally decreased over time in the untreated control, whereas VA promoted them over time. This effect on the Firmicutes could be associated with the VA providing them with an additional nutrient source while also being further metabolized into other potentially bioactive molecules that could be modulating other genera [52]. Concerning other probiotic gram-positive genera, *Bifidobacterium* saw an increase in abundance over time if left without supplementation; however, in all samples treated with phenolic acids, there was an increase in abundance compared to the control at its respective timepoint, with GA showing the most notable improvement. On the other hand, GA treatment saw reduction in the prevalence of the *Clostridium* genus after 24 h and 48 h, with the largest decrease being seen at the later timepoint. Previous studies have evaluated the effects of GA in inhibiting the growth of specific species of *Clostridium* while reporting either no effect or the support of specific species of *Bifidobacterium* [53]. VA treatment has been reported in the past as a potential method for inhibition of *Clostridium*, especially within the context of fermentation [54]. However, other studies have detected phenolic acid resistance genes within other specific *Clostridium* species, which could have played a role that explains how this genus was promoted by VA in the current study [55,56]. Samples treated with PA saw a genus-level relative abundance similar to that of the untreated control at each respective timepoint, while only a slight numerical increase was seen in *Bifidobacterium* and *Lactobacillus*.

Regarding the abundance of the *Escherichia* genus within the Proteobacteria phylum, there was a noticeable difference between the groups treated with GA and VA when compared to the untreated control and group treated with PA. Phenolic acids have long been known for being especially bioactive against gram-negative bacteria for their capability to diffuse freely through the outer membranes of gram-negative bacteria [57]. This outcome is desirable considering that most poultry-borne pathogens are gram-negative Proteobacteria. In addition to this, the abundance of Proteobacteria is considered a marker of dysbiosis and can be induced with common pathogenic gram-negative bacteria while also being able to displace Firmicutes [58]. The balance between the natural antimicrobial potency of each compound and their tolerances to changes in the environment could account for why PA had little effect over the Proteobacteria, whereas GA and VA were more active in modulating the microbiome in general, since the presence and number of specific functional groups has been reported to influence the interaction of specific phenolic acids with gram-negative bacteria [59]. However, the PA-treated samples showed a noticeable reduction in *Acinetobacter*, which is a gram-negative bacterium that has been associated with causing nosocomial infections, and has been reported as having an antibiotic-resistance pattern and negatively affects chicken growth, though GA and VA remained more effective against this genus [60,61]. The negative correlation between *Escherichia* growth and probiotic genera of bacteria such as *Lactobacillus* and *Bifidobacterium* shows an inverse relationship between the abundance of these bacteria, which supports the results found in the current study [62]. Previously published research has also proposed treatment with GA as a potential method for promoting probiotic bacteria and reducing enteric pathogens by way of promoting the inverse relationship that exists between their growth patterns [63].

Analysis of the microbiome at the species level was performed by quantifying the number of species, but also by calculating the alpha diversity using the Shannon Index and Inverse Simpson Index for identifying diversity and evenness within the bacterial species found in each sample, while beta diversity was calculated using Sorenson’s Coefficient, which analyses similarity of microbial populations between samples [28]. These indices have been used as markers for gaining a better understanding of the composition of complex microbial communities and are especially useful when analyzing and interpreting 16S-rRNA gene sequencing data [64]. Microbial diversity in the host GI tract has gained importance in animal production for its potential in improving animal health and yield, as well as making them more resilient to disease, increasing their weight, and reducing the load of foodborne and zoonotic pathogens, though some of the correlations associating specific genera of bacteria with improvements in animal performance remain unclear [65]. With the importance of microbial diversity in mind, there has been increasing concern over the negative effects that interventions involving synthetic antimicrobials and conventional antibiotics have on the microbial composition of the GI tract as these have been shown to cause dysbiosis that makes the host more susceptible to disease and inflammation, but has also been found to alter the gut microbiome in a way that allows for other opportunistic pathogens to colonize the GI tract and remain there long-term, posing an additional threat to food safety [66]. With the importance of microbial diversity in mind, the number of species and their similarities between groups were quantified and compared using three- and four-way Venn diagrams that identified similar OTUs of the core microbiome. Further, beta diversity was evaluated through Sorenson’s Coefficient in which two microbial populations are compared to each other, yielding a quotient between 0 and 1, with values closer to 1 indicating more similarities in the species found between the microbial population being compared and values closer to 0 indicating populations that are more dissimilar to each other [67,68]. Alpha diversity was evaluated through the Shannon Index, which determines species diversity within a given group, while Inverse Simpsons was used for determining evenness, which is a parameter known to be inversely correlated with diversity, meaning that a higher Inverse Simpsons Index denotes less evenness, which is commonly associated with more-diverse communities [69,70].

When analyzing the average number of species found in each group and narrowing them down to a list containing the common OTUs found among replicates, there was a reduction in the number of total OTUs that could be attributed to a given group, which could be explained by the natural variability that exists between reads and collection times [71]. Furthermore, this analysis showed the number of unique OUT species in the control increase over time along with the number of total species identified. At 24 h, all control and treatment groups shared a similar core microbiome. On the other hand, at the 48 h timepoint, control, GA, and PA became more similar, suggesting that at this stage, both compounds were exerting a similar antimicrobial pressure over the same microbes, possibly related to the compounds reaching a similar association state, while VA displayed a different pattern. Further comparison between groups using Sorenson’s Coefficient corroborated that, over time, microbial communities became more dissimilar from those found at 0 h but more similar between more-advanced timepoints. When comparing treatments to the control at their respective timepoints, PA showed more similarity to the control at 24 h as it had a value closer to 1 compared to the GA and VA treatments, while at 48 h, GA and PA were more similar to their respective controls, which is in agreement with the core and unique OTUs detected in the Venn diagrams.

Differences in the Shannon Index for the control at 0 h, 24 h, and 48 h timepoints were not statistically significant but did show a slight decrease as time progressed. A similar pattern was followed by the Inverse Simpson Index, throughout the three timepoints, with the values at 48 h being statistically significant, meaning that the microbiome at this stage was becoming more even or less diverse. These indexes reveal that despite an initial increase in the number of species detected from 0 h to 24 h, only accounting for species number does not represent the actual composition of the microbial community at that time since indexes also consider the amount of hits for each species detected. These results are not unexpected since if left uninterrupted with a fixed amount of nutrients, over time, specific bacteria will out-compete and out-grow others, displacing them and reducing their overall abundance, even if they can still be detected [72]. When analyzing samples within the same timepoint, there was also no statistically significant difference between treatment groups, as compared to the control, but there was a slight numerical increase in the Shannon index of the GA-treated group at 24 h, in addition to a reduction in evenness, as exhibited by a higher Inverse Simpson Index than the control. On the other hand, a slight reduction in diversity was seen in the GA-treated group at the 48 h timepoint, but evenness remained lower than the control at the same timepoint. Samples treated with the other compounds PA and VA at the 48 h timepoint saw a slight increase in diversity and a reduction in evenness, especially in those treated with VA showing the most significant effect in reducing evenness. These results suggest that the compounds tested do not have a significant effect on the alpha indexes for microbial diversity across time, but when using another alpha index, it did reveal an improvement related to evenness, especially at the 48 h timepoint as all treated groups, and most notably VA, were above the control at the same timepoint.

## 5. Conclusions

The use of organic weak acids such as phenolic compounds has emerged as a viable alternative to conventional antibiotics and synthetic antimicrobials as forms of pathogen control, especially for ST in poultry products. This study evaluated their efficacy in a simulated environment using cecal fluid as the medium. Though high doses of these compounds are needed to elicit antimicrobial effects against ST, they can still reduce the overall load of the bacteria and other similar gram-negative pathogens with minimal disruption to the overall integrity of the microbiome in the GI tract. The effects of these compounds can be attributed to their molecular structures, which allow them to have a specific effect on important gram-negative pathogens, but are features that require further study in order to understand how to implement these products efficiently and effectively in an in vivo scenario.

## Figures and Tables

**Table 1:** Antimicrobial potential of phenolic acids in LB with adjusted pH.

pH	Concentration (mg/mL)																																										
		0.016			0.031			0.063			0.125			0.25			0.5			1			1.5			2			2.5			3			3.5			4			4.5		
	LB	G	P	V	G	P	V	G	P	V	G	P	V	G	P	V	G	P	V	G	P	V	G	P	V	G	P	V	G	P	V	G	P	V	G	P	V	G	P	V	G	P	V
4	+	+	+	+	+	+	+	+	+	+	+	+	+	+	+	+	+	+	-	+	+	-	+	-	-	+	-	-	+	-	-	+	-	-	+	-	-	+	-	-	+	-	-
5	+	+	+	+	+	+	+	+	+	+	+	+	+	+	+	+	+	+	+	-	+	+	-	+	+	-	+	+	-	+	-	-	-	-	-	-	-	-	-	-	-	-	-
6	+	+	+	+	+	+	+	+	+	+	-	+	+	-	+	+	-	+	+	-	+	+	-	+	+	-	+	+	-	+	+	-	+	+	-	+	-	-	+	-	-	-	-
7	+	+	+	+	+	+	+	+	+	+	-	+	+	-	+	+	-	+	+	-	+	+	-	+	+	-	+	+	-	+	+	-	+	+	-	+	-	-	+	-	-	-	-
8	+	+	+	+	+	+	+	-	+	+	-	-	+	-	-	+	-	-	+	-	-	+	-	-	+	-	-	+	-	-	-	-	-	-	-	-	-	-	-	-	-	-	-
9	+	+	+	+	+	-	+	-	-	+	-	-	+	-	-	+	-	-	+	-	-	+	-	-	+	-	-	+	-	-	-	-	-	-	-	-	-	-	-	-	-	-	-

Plating samples in LB agar was used to confirm growth (+) or complete inhibition (-) of *Salmonella enterica* serovar Typhimurium (ST) after pH-adjusted treatments with gallic acid (G), protocatechuic acid (P), and vanillic acid (V).

## Data Availability

All data generated in this study will be made available upon request. 16S-rRNA gene sequencing data can be found in NCBI under the BioProject with the number PRJNA797108.
